# Characterization of a novel inhibitor for the New Delhi metallo-β-lactamase-4: Implications for drug design and combating bacterial drug resistance

**DOI:** 10.1016/j.jbc.2023.105135

**Published:** 2023-08-06

**Authors:** James B. Thoden, Bogdan M. Benin, Adam Priebe, Woo Shik Shin, Ramaiah Muthyala, Yuk Yin Sham, Hazel M. Holden

**Affiliations:** 1 Department of Biochemistry, University of Wisconsin, Madison, Wisconsin, USA; 2 Department of Pharmaceutical Sciences, Northeast Ohio Medical University, Rootstown, Ohio, USA; 3 Department of Integrative Biology and Physiology, University of Minnesota, Minneapolis, Minnesota, USA; 4 Department of Experimental & Clinical Pharmacology, University of Minnesota, Minneapolis, Minnesota, USA; 5 Bioinformatics and Computational Biology Program, University of Minnesota, Minneapolis, Minnesota, USA

**Keywords:** antibiotic resistance, antibiotics, computational biology, crystallography, enzyme structure, enzyme kinetics

## Abstract

The bacterial metallo-β-lactamases (MBLs) catalyze the inactivation of β-lactam antibiotics. Identifying novel pharmacophores remains crucial for the clinical development of additional MBL inhibitors. Previously, 1-hydroxypyridine-2(*1H*)-thione-6-carboxylic acid, hereafter referred to as 1,2-HPT-6-COOH, was reported as a low cytotoxic nanomolar β-lactamase inhibitor of Verona-integron-encoded metallo-β-lactamase 2, capable of rescuing β-lactam antibiotic activity. In this study, we explore its exact mechanism of inhibition and the extent of its activity through structural characterization of its binding to New Delhi metallo-β-lactamase 4 (NDM-4) and its inhibitory activity against both NDM-1 and NDM-4. Of all the structure-validated MBL inhibitors available, 1,2-HPT-6-COOH is the first discovered compound capable of forming an octahedral coordination sphere with Zn2 of the binuclear metal center. This unexpected mechanism of action provides important insight for the further optimization of 1,2-HPT-6-COOH and the identification of additional pharmacophores for MBL inhibition.

Without question, the discovery of penicillin in 1928 changed the course of human history. Indeed, it paved the way for the antibiotic revolution beginning in 1942 when penicillin G became widely available (1, 2). The human life expectancy at the beginning of the 20th century was approximately 47 years but rapidly increased to nearly 79 years in the United States during the 1950s to 1970s. This was due in part to the use of β-lactam antibiotics for the treatment of bacterial infections. Unfortunately, the report in 1940 of a penicillin-resistance gene in *Escherichia coli*, was a warning sign of what was to come, namely the development of bacterial resistance to β-lactam therapeutics (2, 3). The continuing emergence of antibiotic resistance has become such a public health threat (https://www.cdc.gov/drugresistance/biggest-threats.html) and a global burden to our health care (4) that the World Health Organization declared November 18 to 24, 2022, to be “World Antimicrobial Awareness Week.”

There are four mechanisms for bacterial resistance: limiting drug uptake, modifying the drug target, inactivating the drug, or removing the drug *via* efflux pumps (5). The β-lactamases function by the drug inactivation mechanism. Specifically, they catalyze the hydrolysis of the amide bond of the β-lactam ring. There are four classes of β-lactamases referred to as A, B, C, and D (6). Enzymes of the A, C, and D classes rely on an active site serine for catalysis, with some members being clinically inhibited by compounds such as clavulanic acid, sulbactam, tazobactam, and avibactam (7, 8). Class B members, known as the metallo-β-lactamases (MBLs), are structurally distinct from the Class A, C, and D enzymes, require one or two zinc ions for catalytic activity, and are not inhibited by currently approved therapeutics (9). Of all the β-lactamase inhibitors currently in clinical trials, taniborbactam, and xeruborbactam are the only candidates capable of inactivating all four classes of β-lactamases (10, 11, 12) with taniborbactam recently completing its phase III clinical trial (NCT03840148). As a consequence of this lack of inhibitors, and in light of their ability to hydrolyze a broad spectrum of β-lactam–based drugs, the Class B enzymes are of utmost concern (13). On the basis of metal content and active site architectures, the MBLs are further divided into three subclasses, B1, B2, and B3, with most belonging to the B1 group to date.

The focus of this investigation is on the MBL referred to as New Delhi metallo-β-lactamase (NDM)-1 (subclass B1), which was first identified in a patient in New Delhi, India in 2009 (14). Strikingly, the enzyme was shown to hydrolyze all known β-lactam antibiotics at the time, with the exception of aztreonam, and bacteria carrying the gene for NDM-1 were subsequently deemed in the mainstream media as “superbugs.” In contrast to other MBLs, NDM-1 is a membrane-bound protein. Importantly, it has experienced a rapid evolution since 2009 with many variants reported to date (15, 16). One variant of significance is NDM-4, first identified in 2012 (17). Although it differs from NDM-1 by a single amino acid substitution, namely M154L, it demonstrates increased enzymatic activity against the carbapenems and several cephalosporins. Of the 44 variants reported thus far, 43% contain the M154L substitution in addition to other amino acid changes (https://www.ncbi.nlm.nih.gov/pathogens/refgene/#ndm).

Once it was determined that gram-negative bacteria carrying NDMs were capable of hydrolyzing meropenem and imipenem, it became clear that a search for inhibitors of these enzymes was critical. These searches included attempts to repurpose existing drugs which led to the discovery of l-captopril, an angiotensin-converting enzyme inhibitor used for the treatment of hypertension and some types of congestive heart failure, as a low micromolar inhibitor of NDM-1 (18). Unfortunately, many of the potential inhibitors tested in these searches functioned by chelating the zincs in the active site, which undoubtedly would lead to significant side effects (19). Importantly, however, it was reported in 2017 that 1-hydroxypyridine-2(*1H*)-thione-6-carboxylic acid, shown in Figure 1 and hereafter referred to as Compound **1**, functions as a potent low cytotoxic nanomolar inhibitor of Verona-integron-encoded MBL 2, another MBL found in clinical isolates of ESKAPE pathogens (20). Analogs of this compound (also referred to as hydroxypyridinethione, HOPTO) have subsequently been shown to exhibit a broad range of inhibition activities against NDM-1 and other zinc metalloenzymes including human carbonic anhydrase II and matrix metallo-proteinase 12 with a wide range of inhibitory and selectivity activities (21). Curious as to whether this “first-in-its-class” compound would also inhibit NDM-4, we initiated a combined biochemical and structural investigation as reported herein. For this analysis, three high-resolution X-ray structures were determined, kinetic and inhibition data were measured, and a computational study was conducted. Taken together our results provide a new and important platform for inhibitor design against the MBLs in general.

**Figure 1:**
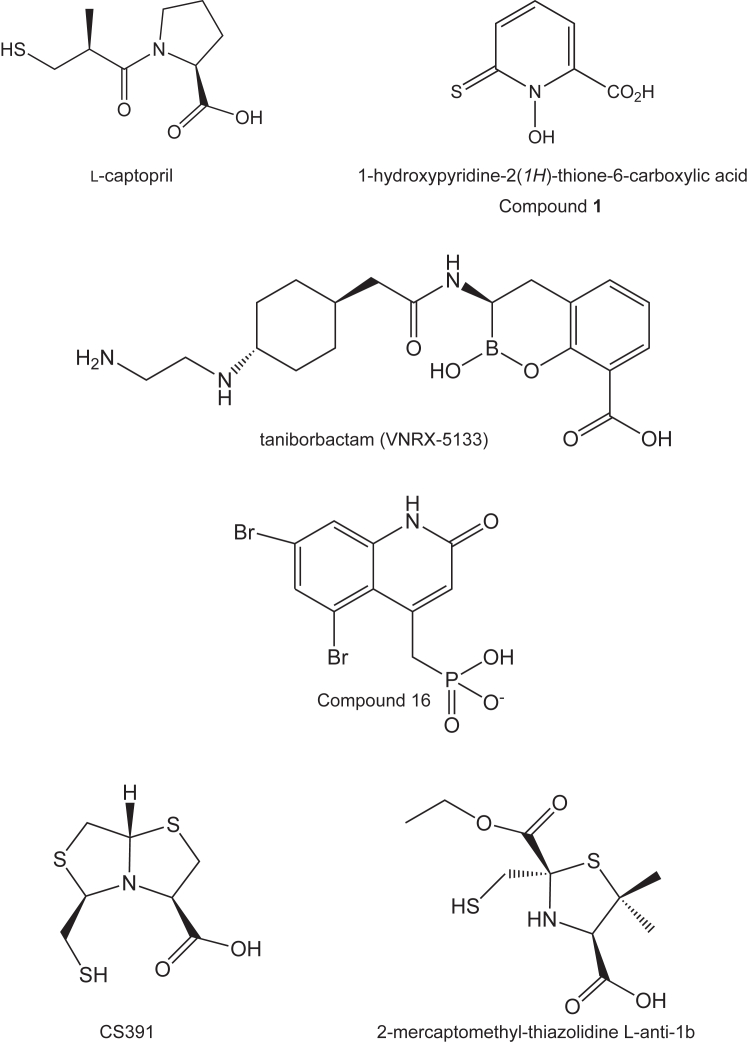
**Inhibitors of New Delhi metallo-β-lactamases.**

## Results

### Structure of apo NDM-4

**Figure 2:**
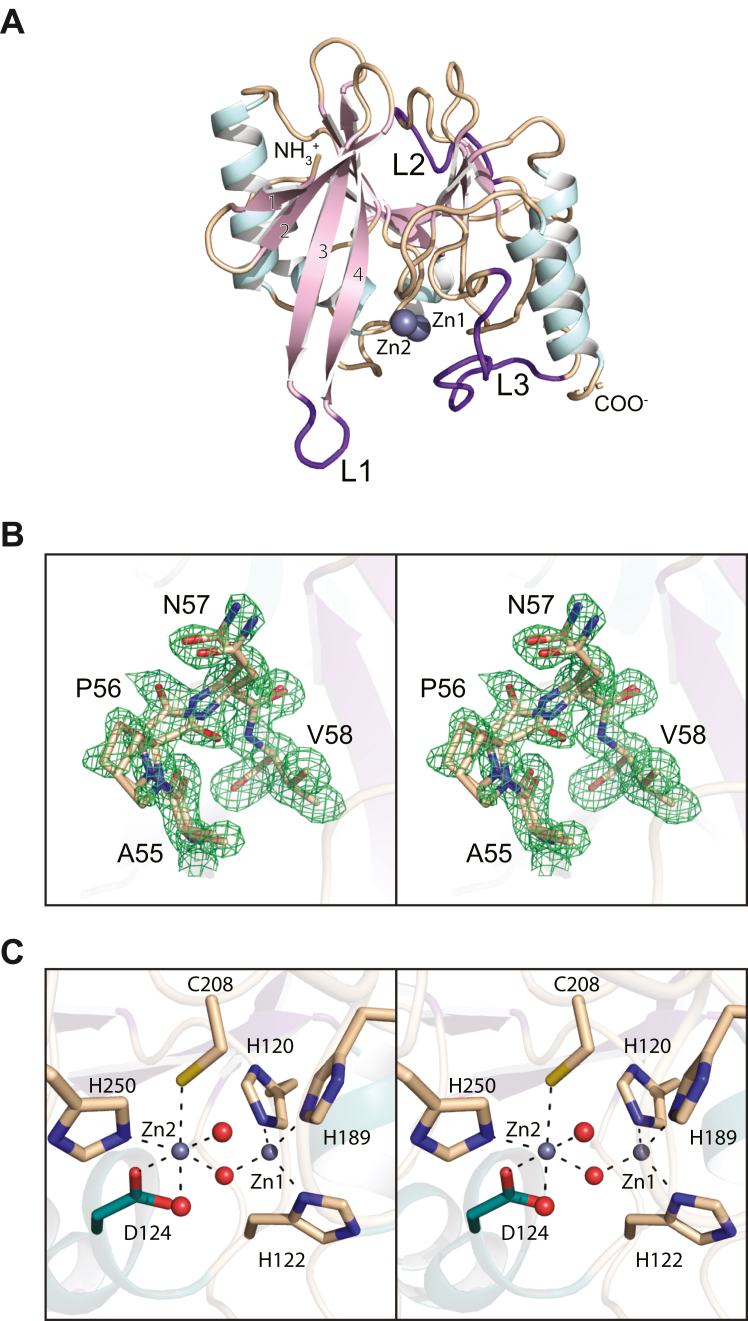
**Structure of NDM-4 (apoenzyme).** A ribbon representation of the monomer is shown in (*A*) with the β-strands and α-helices display in *pink and light blue*, respectively. Loops L1, L2, and L3 are highlighted in *purple*. A stereo view of the electron density corresponding to the region defined by Ala 55 to Val 58 is presented in (*B*). The map was calculated with (2*F*_o_-*F*_c_) coefficients and contoured at 1σ. As can be seen, this region adopts two distinct reverse turn conformations. A close-up stereo view of the binuclear metal center is provided in (*C*). Metal:ligand bonds are indicated by the *dashed lines*. Ordered solvent molecules are depicted as *red spheres*. This figure and Figures 3 and 4 and Figure 7, Figure 8, Figure 9 were prepared with PyMOL (60) (https://pymol.org/2/). NDM, New Delhi metallo-β-lactamase.

The first crystals of NDM-4 utilized in this investigation were grown at room temperature using poly(ethylene glycol) 3350 as the precipitant (pH 6.0). They belonged to the space group *P*2_1_ with a monomer in the asymmetric unit. The model of the enzyme was refined to an overall *R*-factor of 17.9% at 1.3 Å resolution. Relevant X-ray data collection and model refinement statistics for all structures are provided in Table 1. The electron density for the polypeptide chain was continuous from Gly 42 to Arg 270. A ribbon representation of the enzyme is provided in Figure 2*A*. Its three-dimensional architecture is dominated by an eight-stranded mixed β-sheet, a four-stranded mixed β-sheet, and five α-helices with the second helix, defined by Gln 123 to Ala 134, distorted by the presence of Gly 127 and Gly 128. The reverse turn connecting β-strands 2 and 3 (Ala 55 to Val 58) adopts two distinct conformations as shown in Figure 2*B*.

**Table 1:** X-ray data collection statistics and model refinement statistics

	NDM-4 apoenzyme	NDM-4/l-captopril complex	NDM-4/Compound 1
Resolution limits (Å)	50–1.3 (1.4–1.3)^a^	50–1.3 (1.4–1.3)^a^	50–1.3 (1.4–1.3)^a^
Number of independent reflections	46,573 (9062)	91,595 (16,761)	44,587 (7394)
Completeness (%)	98.3 (96.4)	95.4 (88.0)	92.7 (77.5)
Redundancy	5.2 (2.8)	3.4 (1.9)	4.8 (1.4)
avg I/avg σ(I)	16.1 (3.1)	10.4 (2.5)	16.5 (2.3)
*R*_sym_ (%)^b^	5.1 (34.1)	5.7 (29.0)	4.9 (28.6)
c*R*-factor (overall)%/no. reflections	17.9/46,573	20.0/91,595	20.3/44,587
*R*-factor (working)%/no. reflections	17.8/44,388	19.9/86,986	20.2/42,417
*R*-factor (free)%/no. reflections	19.9/2235	22.3/4609	22.4/2170
Number of protein atoms	1742	3541	1777
Number of heteroatoms	235	558	250
Average B values			
Protein atoms (Å^2^)	10.8	11.0	15.8
Ligand (Å^2^)	12.9	20.9	21.6
Solvent (Å^2^)	20.6	21.6	23.6
Weighted RMSDs from ideality			
Bond lengths (Å)	0.007	0.008	0.007
Bond angles (º)	1.43	1.47	1.36
Planar groups (Å)	0.008	0.008	0.007
Ramachandran regions (%)^d^			
Most favored	98.2	98.7	98.7
Additionally allowed	1.8	1.3	1.3
Generously allowed	0.0	0.0	0.0
PDB code	8SK2	8SKO	8SKP

a Statistics for the highest resolution bin.

b *R*_sym_ = $\left(\sum |\text{I}\;-\;\overset{\text{-}}{\text{I}}|/\sum \;\text{I}\right)$ x 100.

c *R*-factor = (Σ|*F*_o_-*F*_c_|/Σ|F_o_|) x 100, where *F*_o_ is the observed structure-factor amplitude and *F*_c_ is the calculated structure-factor amplitude.

d Distribution of Ramachandran angles according to PROCHECK (61).

A close-up stereo view of the active site is provided in Figure 2*C*. The two zincs that form the binuclear metal center are separated by 3.5 Å. All of the side chains involved in metal coordination reside on random coil regions with the exception of Asp 124, which is positioned at the start of an α-helix. Zn1 is ligated by the side chains of His 120, His 122, His 189 at the so-called “histidine” site and a bridging water (or hydroxide ion) in a distorted tetrahedral arrangement. The bond lengths between Zn1 and the imidazole nitrogens of His 120, His 122, and His 189 are 2.0, 2.1, and 2.0 Å, respectively, whereas the distance between Zn1 and the bridging water is 1.8 Å. Zn2 is surrounded in an octahedral coordination sphere provided by the side chains of Asp 124, Cys 208, His 250 at the so-called “cysteine” site, the bridging solvent and two water molecules. The metal-ligand distances are all ∼2 Å with the exception of that formed between the zinc ion and the thiolate of Cys 208, which is 2.5 Å. In the model, O^δ2^ of Asp 124 lies at 2.1 Å from Zn2, whereas O^δ1^ sits at 2.7 Å from the bridging solvent of the binuclear metal center.

### Structure of the NDM-4/(2S)-1-(3-mercapto-2-methylpropionyl)–l-proline complex (l-captopril)

The crystals of the NDM-4/l-captopril complex utilized for this analysis were also grown from poly(ethylene glycol) 3350 and belonged to the *P*2_1_ space group. This time, however, there were two monomers in the asymmetric unit. The model of the NDM-4/l-captopril complex was refined to an overall *R*-factor of 20% at 1.3 Å resolution. The electron densities for both polypeptide chain backbones were continuous from Gly 41 to the C terminus and their α-carbons correspond with a RMSD of 0.3 Å. Given their similarities, and for the sake of clarity, the following discussion refers to subunit B in the X-ray coordinate file unless otherwise indicated.

**Figure 3:**
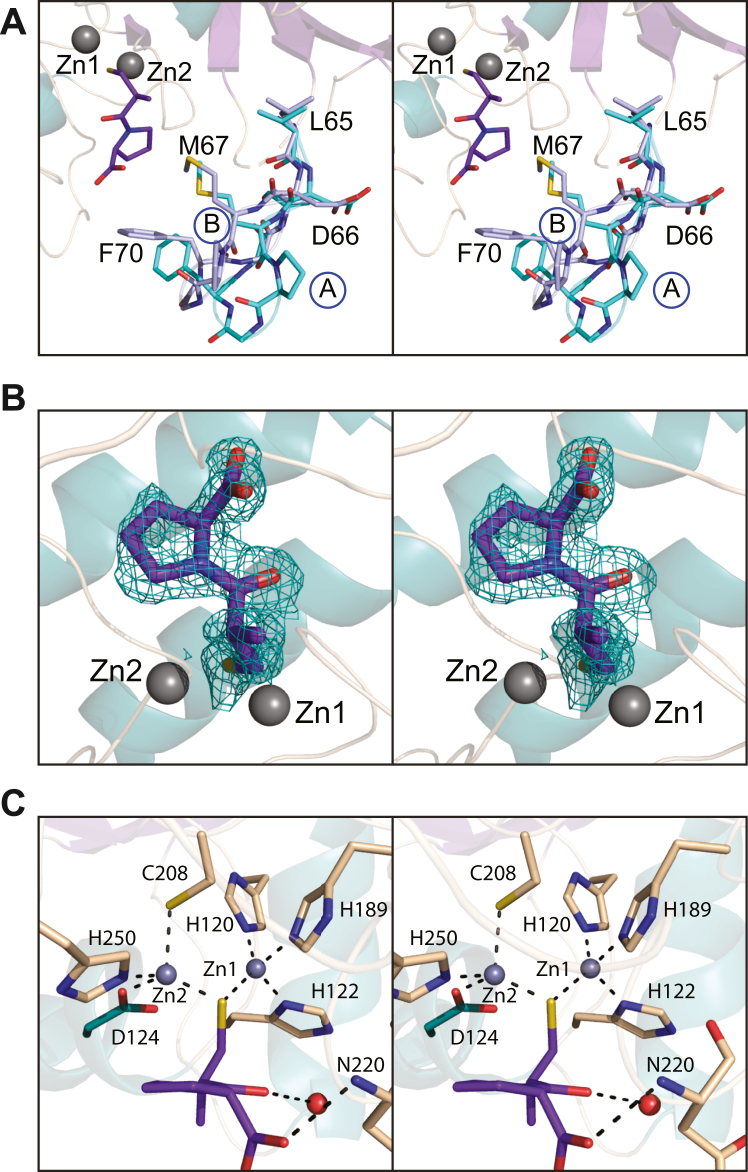
**Structure of the NDM-4/****l****-captopril complex.** The region defined by Leu 65 to Met 67 adopts two distinct conformations in the NDM-4/l-captopril complex as shown in stereo in (*A*). The two conformations are labeled *A* and *B*. The observed electron density corresponding to l-captopril is presented in stereo in (*B*). The map shown was calculated with (*F*_o_-*F*_c_) coefficients and contoured at 3σ. The inhibitor, l-captopril, was not included in the X-ray coordinate file used to calculate the omit map, and thus there is no model bias. A close-up stereo view of the binuclear metal center with bound l-captopril is provided in (*C*). Metal:ligand bonds are indicated by the *dashed line*. The bound l-captopril is colored in *purple bonds*. NDM, New Delhi metallo-β-lactamase.

The α-carbons for the apoenzyme and the NDM-4/l-captopril complex superimpose with a RMSD of 0.4 Å. The reverse turn in the apoenzyme structure that adopts two distinct conformations (Ala 55 to Val 58) is a distorted Type I turn in the NDM-4/l-captopril model. In both subunits of the NDM-4/l-captopril model, the region delineated by Leu 65 to Ala 72 adopts two distinct conformations, labeled A and B as shown in Figure 3*A*. Only conformation A is observed in the apoenzyme model. Conformations A and B in the NDM-4/l-captopril complex result from slightly differing backbone torsional angles initiating at Leu 65, which taken together, results in the α-carbons for Pro 68, Gly 69, and Phe 70 differing in space by 3.4 Å, 3.9 Å, and 2.5 Å, respectively. In conformation B, C^ζ^ of Phe 70 lies within 3.6 Å of a carboxylate oxygen of the l-captopril ligand. In conformation A, this distance is 6 Å.

The electron density corresponding to the bound l-captopril is presented in Figure 3*B*. The sulfur of the ligand lies within 2.2. Å and 2.1 Å of Zn1 and Zn2, respectively. The bridging solvent in the apoenzyme model, as well as three additional waters are extruded from the vicinity of the binuclear metal center upon l-captopril binding. As a consequence, the coordination geometry surrounding Zn2 changes from octahedral to distorted tetrahedral shown in Figure 3*C*. Metal:ligand bond lengths for both zinc ions in the binuclear metal center lie between 2.0 Å and 2.4 Å. In addition to its interaction with the binuclear metal center, the ligand also lies within hydrogen bonding distance to an ordered water molecule and the backbone amide nitrogen of Asn 220.

### Structure of NDM-4/Compound **1**

Crystals of the NDM-4/Compound **1** complex were obtained from poly(ethylene glycol) 3350 solutions at pH 6.0 and contained one molecule per asymmetric unit. The model was refined to an overall *R*-factor of 20.3% at 1.3 Å resolution. Like that observed for the NDM-4/l-captopril complex, the reverse turn defined by Ala 55 to Val 58 adopted a single distorted Type I turn. Also, as observed in the NDM-4/l-captopril complex, the region delineated by Leu 65 to Ala 72 adopted two distinct conformations. The α-carbons for the NDM-4/Compound **1** and the NDM-4/l-captopril complex models correspond with a RMSD of 0.3 Å.

**Figure 4:**
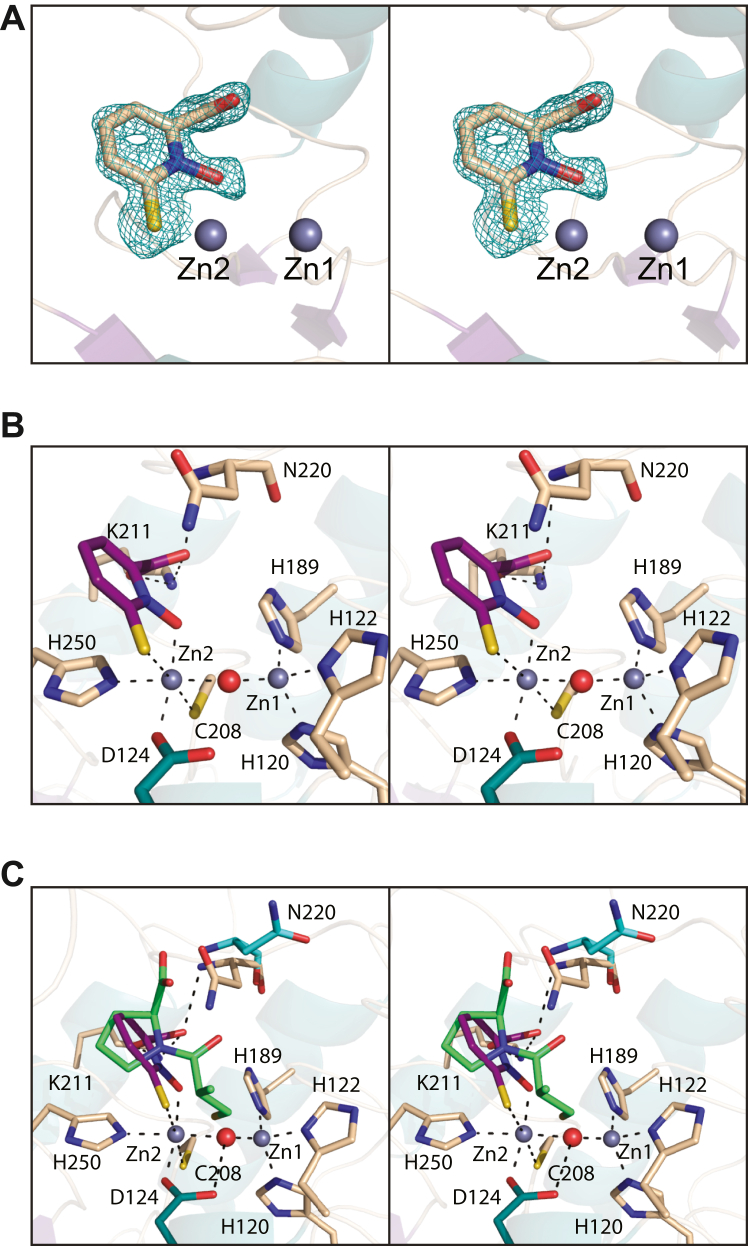
**Structure of the NDM-4/Compound 1 complex.** The observed electron density for the inhibitor is shown in stereo in (*A*) and contoured as described in Figure legend 3. A close-up view of the binuclear metal binding region is presented in stereo in (*B*) with the inhibitor highlighted in *purple bonds*. A superposition of the binding modes for l-captopril and Compound **1**, displayed in *greenand purple bonds* respectively, is provided in (*C*). NDM, New Delhi metallo-β-lactamase.

Shown in Figure 4*A* is the observed electron density associated with the bound ligand, and the immediate vicinity surrounding the binuclear metal center is displayed in Figure 4*B*. Compound **1** functions as a bidentate ligand to Zn2, which is also surrounded by the side chains of Asp 124, Cys 208, and His 250 and the bridging solvent in an octahedral coordination sphere. The bond distances between Zn2 and the sulfur and oxygen atoms of Compound **1** are 2.5 Å and 2.0 Å, respectively. The coordination geometry around Zn1 remains tetrahedral with the side chains of His 120, His 122, and His 189, and the bridging solvent serving as ligands. Both the side chain of Lys 211 and backbone amide nitrogen of Asn 220 serve to anchor the carboxylate moiety of Compound **1** into the active site.

A comparison of the NDM-4–binding modes for l-captopril and Compound **1** is provided in Figure 4*C*. In addition to the difference in coordination geometry to the binuclear metal center, the side chain of Asn 220 swings toward the active site upon Compound **1** binding.

### Kinetic analyses

To ensure the purified NDM-1 and NDM-4 (M154L) were enzymatically active, their kinetic parameters were determined based on steady state kinetics for the hydrolysis of nitrocefin (Fig. S1) (20, 22, 23). Nitrocefin is a well-known chromogenic cephalosporin, whose degradation can be readily followed and quantified spectroscopically. For NDM-1, the determined *K*_M_ and *k*_cat_ values were 10.9 ± 0.6 μM and 86 ± 11 s^−1^, respectively, with a catalytic efficiency (*k*_cat_/*K*_M_) of 7.9 × 10^6^ M^−1^ s^−1^. For NDM-4, the determined *K*_M_ and *k*_cat_ values were 9.4 ± 0.2 μM and 70 ± 7 s^−1^, respectively, with a catalytic efficiency of 7.4 × 10^6^ M^−1^ s^−1^. The determined parameters are in agreement with previous studies (24, 25, 26), indicating that the enzymatic activity, at least toward nitrocefin, remains similar between both enzymes.

The inhibitory activity of Compound **1** was determined *via* a dose-dependent assay using nitrocefin and was compared to l-captopril as the control. The *K*_i_ and IC_50_ of both compounds against NDM-1 and NDM-4 are provided in Table 2. The determined *K*_i_ for Compound **1** was 0.08 μM and 0.12 μM with a corresponding ligand efficiency (LE) of 0.89 and 0.87 against NDM-1 and NDM-4, respectively. The determined *K*_i_ for l-captopril was 164 μM and 210 μM with a corresponding LE of 0.37 and 0.36 against NDM-1 and NDM-4, respectively. Alternative methods (27, 28) for evaluating *K*_i_ are within an order of magnitude difference. The determined *K*_i_ of l-captopril is in agreement with previously reported inhibitory activities against NDM-1 (29). Overall, these are the first reported inhibitory activities of Compound **1** against the NDMs. Comparatively, Compound **1** demonstrates more than a 1600-fold potency over l-captopril with a 2.4 times improved LE.

**Table 2:** Inhibitory activities against NDMs

Compound	N	NDM-1			NDM-4		
		*K*_i_ (μM)	IC_50_ (μM)	LE	*K*_i_ (μM)	IC_50_ (μM)	LE
l-captopril	14	164	215	0.37	210	238	0.36
Compound **1**	11	0.08	0.48	0.89	0.12	0.50	0.87

Ligand efficiency (LE) = −1.38 log (*K*_i_)/N where N = number of heavy atoms (62).

### Computational studies

**Figure 5:**
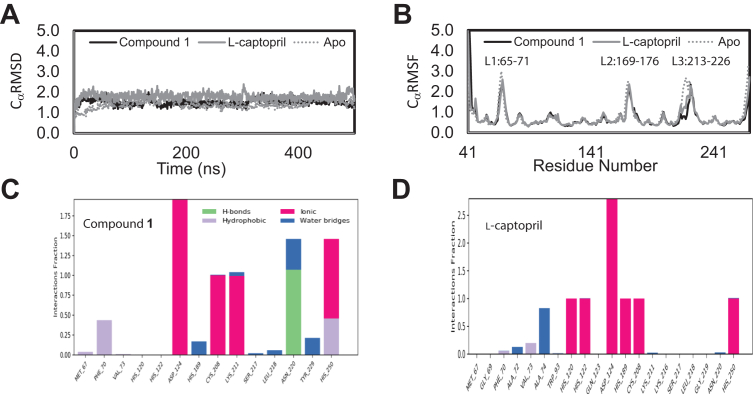
**Molecular dynamics simulation analysis.** (*A*) C_α_RMSD and (*B*) C_α_RMSF plots of NDM-4 in the apo unbound state (*gray dotted line*) and in the bound states with Compound **1** (*black solid line*) and l-captopril (*gray solid line*) over the course of the simulation with the largest fluctuation observed in the L1, L2, and L3 regions. The protein–ligand interaction histograms between NDM-4 with (*C*) Compound **1** and (*D*) l-captopril are shown to highlight the specific molecular interaction (ionic, hydrogen bond, hydrophobic, and water bridges) required for binding. The interaction fraction is the average number of atomic contacts between the ligand and the protein residue over the course of the simulation. NDM, New Delhi metallo-β-lactamase; RMSF, root-mean-square fluctuation.

Molecular dynamics (MD) simulations were carried out for all three solved structures to explore the conformation dynamics of NDM-4 and the role of the loop regions during ligand binding to Compound **1** and l-captopril. The C_α_RMSD and the C_α__RMSF_ are shown in Figure 5, *A* and *B*. The average C_α_RMSD for the apo, Compound **1**, and l-captopril bound NDM-4 were 1.4, 1.5 and 1.8 Å, respectively. The maximum structural fluctuations of the protein backbone were observed at three distinct loop regions referred to as L1, L2, and L3 and delineated by residues Leu 65 to Gly 71, Val 169 to Asn 176, and Ser 213 to Thr 226 with the apo structure exhibiting slightly larger movement as compared to the other two bound states between 2.5 to 3.0 Å. The positions of these loops can be seen in Figure 2*A*. For Compound **1** and l-captopril, the fluctuation at the Ser 213 to Gly 219 region is slightly restricted in L3 loop.

Both the L1 and L3 loops are located at the active site and are involved in ligand binding. Interaction diagrams that monitor the fractional contact of bound ligand with NDM-4 over the course of the simulations are shown in Figure 5, *C* and *D*. Key differences in the nature of the molecular interactions can be observed between Compound **1** and l-captopril. Compound **1** binds strictly to Zn2 at the cysteine site (Asp 124, Cys 208, and His 250) with direct hydrophobic interactions between its aromatic core and residues Met 67 and Phe 70 in the L1 loop. Throughout the entire course of the simulation, the carboxylic acid group of Compound **1** is engaged in a salt bridge interaction with the side chain of Lys 211 and in a hydrogen bond with the amide hydrogen of Asn 220. Additional water bridge interactions can be observed between Compound **1** and L3, involving Ser 217, Leu 218, Tyr 229 and, in particular, Asn 220. For l-captopril, which binds as a bridge-chelate between Zn1 and Zn2, hydrophobic interactions between its methyl group and Phe 70/Val 73 in the L1 loop are observed. The water–bridge interaction observed at Ala 72 and Ala 74, whose side chains are positioned on the opposite side of the L1 loop, is mediated *via* their backbone amide groups. As its carboxylic group is pointing away from the L3 loop, no significant contact occupancy was observed even though its nearest atom is within 4 Å.

**Figure 6:**
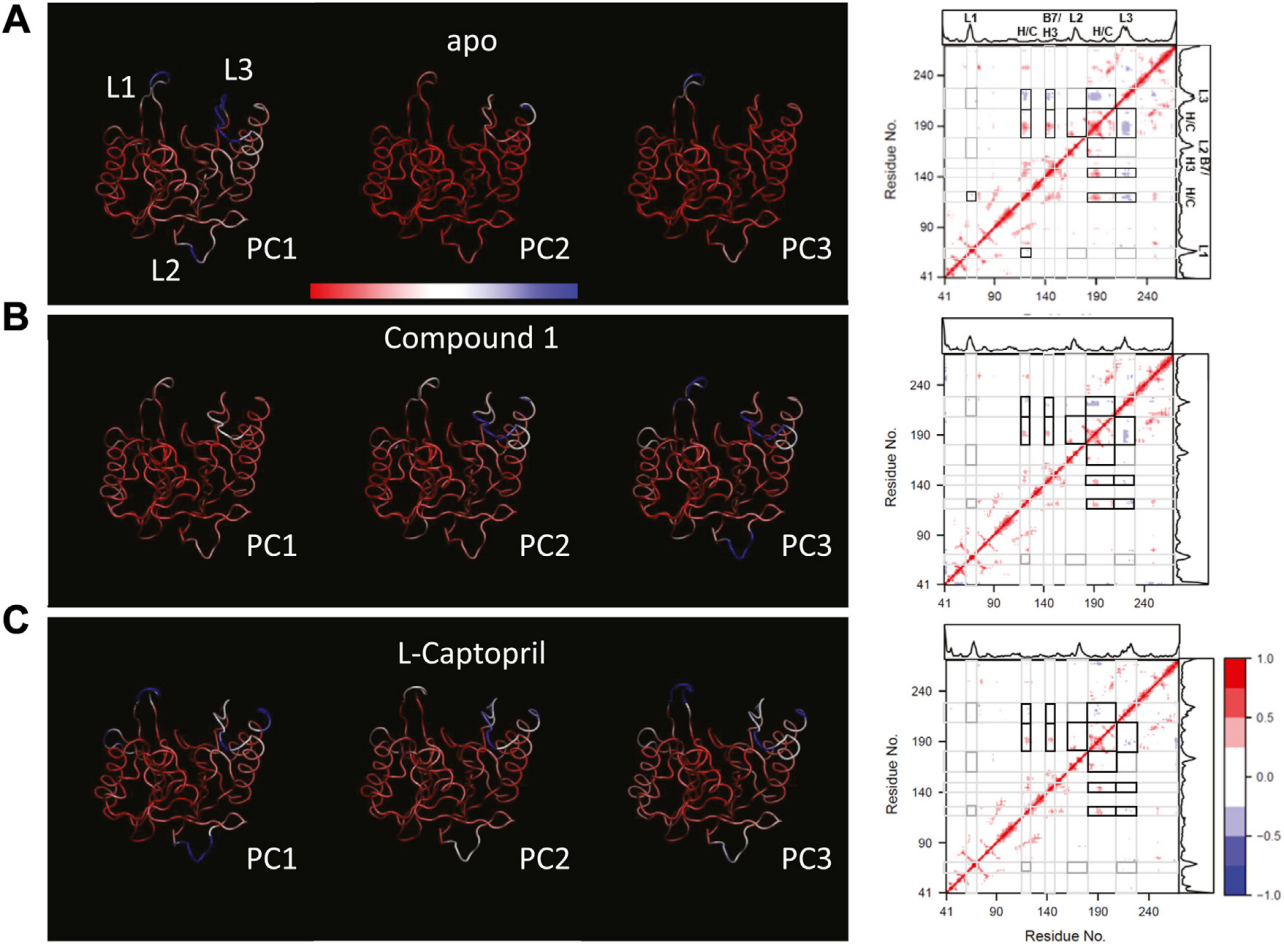
**Principal component and dynamics cross-correlation analyses.** The top three principal components of motion for the (*A*) apo unbound, (*B*) Compound 1 bound and (*C*) l-captopril bound states of NDM-4 over the course of the simulations are shown as PC1, PC2, and PC3 with the regions showing the smallest (*red*) and largest (*blue*) movement. The dynamics cross correlation showing positively correlated (*red*), negatively correlated (*blue*), and uncorrelated (*white*) regions of NDM-4. Both correlated and anticorrelated regions are highlighted *black boxes*. The uncorrelated regions are highlighted in *gray boxes*. NDM, New Delhi metallo-β-lactamase; PC, principal component.

To further explore the nature of protein dynamics involved in ligand binding, principal component analyses (PCA) and dynamic cross-correlation analyses (DCCA) were carried out. The three dominant motions identified account for 32.1%, 42.7%, and 26.9% of the total motion for the apo, Compound **1**, and l-captopril bound NDM-4, respectively. The opening and closing of the active site can be observed with the movement of L1 and L3 in all three structures with Compound **1**–bound NDM-4 showing the least movement (Fig. 6*A* and Supplementary movie files). Although concurrent movement of all three loops can be observed in at least one of the three identified principal components of motion for each of the three structures, DCCA did not show any direct correlation among the three loop regions (Fig. 6*B*). The movement of the L1 and L3 loop is directly anticorrelated to the movement of the histidine and cysteine (H/C) zinc-binding sites, whereas the movement of the distal L2 loop is correlated to the same sites in the unbound state. Interestingly, the region consisting of residues Tyr 140 to Pro 150 containing β-strand 7 and α-helix 3 (B7/H3) exhibits strong correlation to the L3 and the H/C sites. Upon ligand binding, the number of residues associated with these correlations are significantly reduced with the complete loss of correlation between the L1 and the H/C sites.

## Discussion

**Figure 7:**
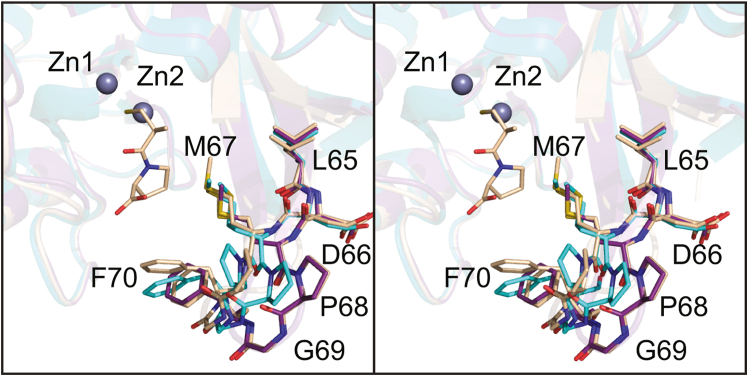
**Comparison of the NDM-4 model presented here to that deposited under PDB accession code****5WIG****.** As expected, the two NDM-4 models are nearly identical with the exception of the loop defined by Leu 65 to Phe 70. The three loop conformations shown here in stereo correspond to our model of the apoenzyme (*purple*), the coordinates deposited in the Protein Data Bank (aquamarine), and the structure of NDM-4/l-captopril complex (*wheat*). The l-captopril ligand is displayed in *wheat bonds*. NDM, New Delhi metallo-β-lactamase.

As reported in 2017, the first three-dimensional model for the apoenzyme of NDM-4 was determined with crystals grown at pH 6.5 from 20% poly(ethylene glycol) monomethyl ether 550 (30). The crystals contained two monomers per asymmetric unit. The model was determined at 1.4 Å resolution and refined to an *R*-factor of 20.0%. Not surprisingly, the α-carbons for the model described here superimpose onto those deposited in the Protein Data Bank (accession code 5WIG) with a RMSD of 0.4 Å. There are several differences, however. The first difference resides in the loop defined by Ala 55 to Val 58. As mentioned above, this loop adopts two distinct conformations in the model reported here. In the coordinates under the PDB accession code 5WIG, the reverse turn adopts only the Type I conformation. Also, as discussed above and illustrated in Figure 3*A*, the region defined by Leu 65 to Ala 72 adopts two conformations labeled A and B in the NDM-4/l-captopril. In our apoenzyme model, only conformation A is observed, but in the model deposited under PDB accession code 5WIG, two conformations are observed, one close to conformation A and the other somewhere between conformations A and B as highlighted in Figure 7. These alternate conformations may be a result of crystalline packing interactions but clearly are not related to the presence or absence of a bound substrate or analog in the active site.

**Figure 8:**
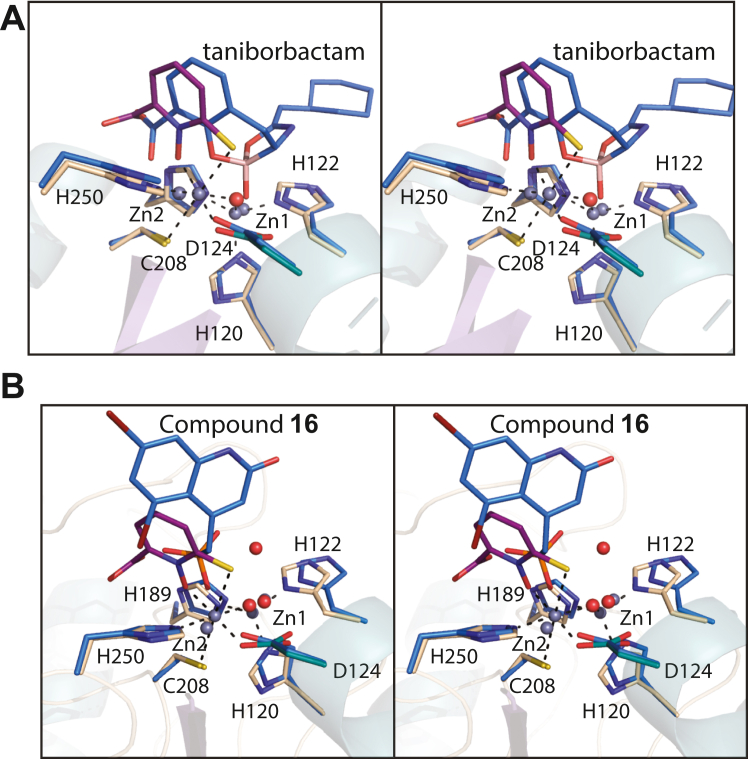
**Comparison of the binding mode of Compound 1 to NDM-4 *versus* transition state analog mimics.** Shown in (*A*) in stereo is a superposition of the NDM-4/Compound **1** and the NDM-1/taniborbactam complex models. The NDM-4 model is highlighted in *wheat bonds* and Compound **1** is colored in *purple bonds*. The NDM-1/taniborbactam complex is presented in *blue bonds*. The bridging water of the binuclear center is depicted as a *red sphere*. A stereo superposition of the NDM-4/Compound **1** and the NDM-1/Compound **16** complex models is shown in (*B*) with the same color coding as described in (*A*). In both (*A*) and (*B*), the coordination geometry about Zn1 and Zn2 in the NDM-4 model is indicated by the *dashed lines*. NDM, New Delhi metallo-β-lactamase.

Given that there are no currently available marketed therapeutics directed against the MBLs, there is a pressing need for the development of such compounds. Rapid progress in the synthesis of boronic acid–based inhibitors has been reported, however, which are designed to target both the serine- and zinc-dependent enzymes (29, 31). Excitingly, one such compound, taniborbactam (VNRX-5133), in combination with cefepime, is now being evaluated in healthy adult subjects for safety and pharmokinetic properties (10, 32). The molecular structure of taniborbactam is provided in Figure 1, and its mode of binding to NDM-1 was reported in 2019 (11). A superposition of the NDM-1 active site with bound taniborbactam (PDB accession code 6RMF) onto the NDM-4 active site harboring Compound **1** is displayed in Figure 8*A*. Interestingly, the binding of taniborbactam to NDM-1 increases the Zn1-Zn2 distance from ∼3.6 Å to 4.3 Å. Within experimental error, the distance between Zn1 and Zn2 when Compound **1** binds remains the same as observed in the apoenzyme model (∼3.6 Å). As can be seen in Figure 8*A*, the boron-bound oxygen of taniborbactam displaces the bridging water (or hydroxide) from the binuclear metal center. In both complexes, the coordination geometry about Zn1 remains tetrahedral. The geometry about Zn2 is octahedral when Compound **1** is bound but is approximately trigonal bipyramidal in the case of taniborbactam.

In addition to the design of boronic acid–based inhibitors, heteroaryl phosphonate compounds have also been examined for their pharmacological activities against both the serine-β-lactamases and the MBLs (33). In the study reported by Pemberton *et al.* (33), 16 compounds were synthesized and tested. Shown in Figure 8*B* is a superposition of the active sites for NDM-1 and NDM-4 with bound Compound **16** (PDB accession code 6NY7) and Compound **1**, respectively. Compound **16**, shown in Figure 1, proved to be the most potent lead compound in their study. As in the case of taniborbactam binding, the distance between the two zinc ions increases to 4.6 Å in the presence of Compound **16**. Whereas the binding of taniborbactam displaces the bridging water (or hydroxide) from the coordination sphere of the binuclear metal center, in the case of Compound **16**, the water is retained, albeit no longer equally distanced from the two zincs (1.9 Å and 3.2 Å to Zn1 and Zn2, respectively). In the NDM-4/Compound **1** complex, the bridging water is within 1.9 Å and 2.0 Å from Zn1 and Zn2, respectively.

**Figure 9:**
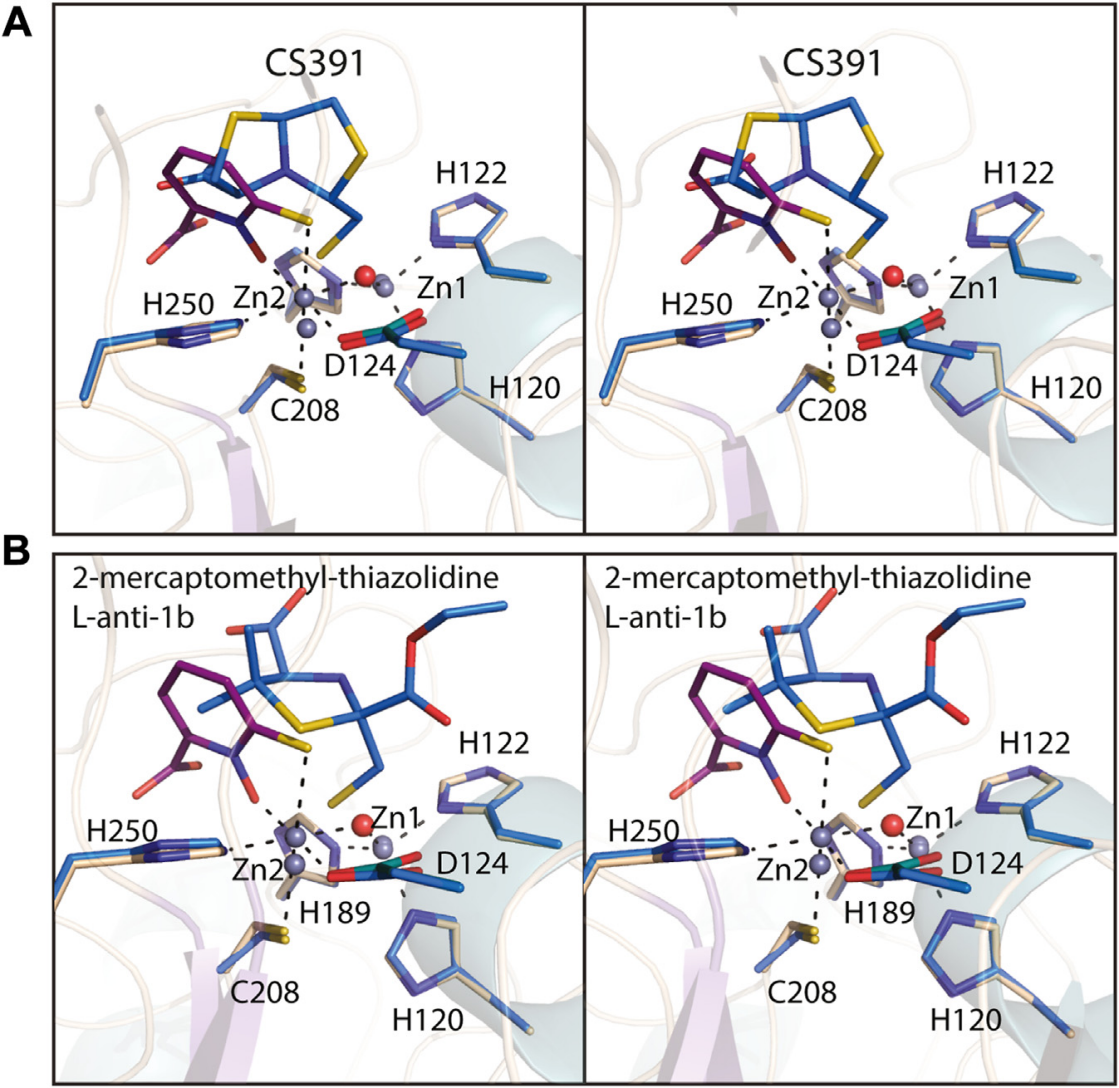
**Comparison of the binding mode of Compound 1 to NDM-4 *versus* those observed with thiolate-based inhibitors to NDM-1.** A stereo superposition of the NDM-4/Compound **1** and the NDM-1/CS391 complexes is presented in (*A*). Likewise, a stereo superposition of the NDM-4/Compound **1** and NDM-1/2-mercaptomethyl-thiazolidine L-anti-1b, 4u41 displayed in (*B*). The color coding is as described in Figure legend 5. NDM, New Delhi metallo-β-lactamase.

In contrast to the boronic acid–based and phosphonate compounds that are designed to mimic the tetrahedral transition state, bisthiazolidines have also been investigated as substrate analogs to specifically inhibit the MBLs (34, 35). The binding of one such inhibitor, CS319 (Figure 1) in complex with NDM-1, has been determined (PDB accession code 4U4L). A superposition of that model onto the NDM-4/Compound **1** coordinates is displayed in Figure 9*A*. In the case of CS391 binding, the bridging water (or hydroxide) is displaced but the Zn1-Zn2 distance remains, within experimental error, the same (∼3.8 Å) as observed in the unliganded NDM-1 structure. In the NDM-1/CS319 complex, both zincs assume tetrahedral ligation spheres.

A series of 2-mercaptomethyl-thiazolidines, designed to mimic reaction intermediates or hydrolysis products of the MBLs, were recently reported (36). A superposition of the structure of NDM-1 in complex with one such compound, 2-mercaptomethyl-thiazolidine L-anti-1b (Figure 1 and PDB accession code 4U4L), onto the NDM-4/Compound **1** model is presented in Figure 9*B*. The thiolate of the inhibitor displaces the bridging water (or hydroxide) and lies equidistant from the zinc ions, both of which adopt tetrahedral coordination spheres. To the best of our knowledge, however, none of the MBLs inhibitors identified thus far, other than Compound **1** presented here, coordinates only to Zn2.

Protein dynamics play an essential role in conformation sampling and in the transition between the unbound “open” and the bound “closed” states. Ligand binding selects and stabilizes the “closed” state which enhances their conformation population (37, 38), making experimental interrogation and structure determination possible (39, 40). In this study we employed MD simulation using our high-resolution X-ray structures to understand the nature of protein dynamics and the molecular forces governing Compound **1**’s potent inhibition as compared to l-captopril. MD simulation in explicit solvent provides a means to capture the dynamic movement of the protein to better understand the variation in the structural changes that cannot be observed from static X-ray crystallographic structures. It also can capture essential solvent interactions that can be missed from these static X-ray models. Indeed, it is complementary and works in parallel with X-ray crystallographic studies to provide a holistic view of the protein-ligand binding process. Previous MD simulation was carried out using homology models based on NDM-1 (41, 42). Our simulations identified the high fluctuating L1, L2, and L3 regions and the essential interactions in the active site involved in ligand binding. Our principal component (PC) analyses showed for the first time the predominant movement of NDM-4 consisting of these three regions. In the unbound state, the “opening” and “closing” of the catalytic site whereby L1 and L3 moved in and out over the H/C zinc–binding sites can be observed primarily in PC1 (Fig. 6). In the Compound **1** and l-captopril bound states, however, these movements can be observed to disperse across all three PCs.

In the past, extensive site-directed mutagenesis studies have been conducted to address the roles of residues required for catalysis (43). There are limited experimental reports examining the dynamical nature of the L2 and L3 regions. However, the importance of L3 was shown in NDM-1 by site-directed mutagenesis (44) which demonstrated that disruption of its hydrophobic core, consisting of Leu 218 and Leu 221 in the L3 region and nearby Leu 269 and Tyr 229, results, in most cases, decreased protein stability with a lowered melting temperature (T_m_) and a significant decrease in the *k*_cat_ values for the L218T and L221T variants. The observed effect is not surprising given that the conformation population of the closed state involving L3 is dependent upon overall protein stability, and the disruption of the L3 hydrophobic core likely alters the preorganization of the active site required for substrate binding and enzyme catalysis (45).

The ability of L1 to transition between the open and closed states has been previously explored spectroscopically in NDM-1 by Crowder and co-workers by determining the relative distance between two spin-labeled residues (46). The study showed the transitional movement of the L1 region occurs in a millisecond timescale during substrate binding and is capable of returning to its original open position after hydrolysis. This agrees with the observed structural difference in the L1 region between the bound and unbound X-ray structures of NDM-4.

Given the different binding modes, the observed molecular interaction fingerprint profiles between Compound **1** and l-captopril were noticeably distinct against NDM-4. One unique design feature of Compound **1** is its ability to form a salt bridge with the evolutionary conserved Lys 211 that is required for anchoring the C3 carboxylate group of all β-lactam antibiotics into the active site (47). It has previously been shown to enhance its inhibition activity over its parent scaffold, 1,2 hydroxy pyrithione, by more than 10-fold against Verona-integron-encoded MBL 2 (20). Similarly, d-captopril, a diastereomer of l-captopril that is capable of forming a similar salt-bridge with Lys 211, also exhibits a 7.8-fold increase in potency against NDM-1 (48).

One unexpected finding in our MD simulation study was the identification of the water bridge interaction between the Compound **1**’s carboxylate group with the side chains of Asn 220 and Tyr 229. Its ability to form a hydrogen bond with the amide backbone of Asn 220 has previously been reported (47). The presence of a water bridge interaction can be determined by X-ray crystallographic studies if high occupancy water is present. In our all-atom MD simulation study in an explicit water system, this was determined by monitoring the average occupancy of ordered waters nearby the bound ligand. Given the negative charged nature of the carboxylate group, the water bridge interaction is a result of hydrogen bonding between the carboxylate group of Compound **1** and the polar side chains of Asn 220 and Tyr 229 that is mediated by water molecules.

For l-captopril, the nature of the interactions required for binding is its metal chelation to the H/C zinc–binding sites, its hydrophobic interaction with the Phe 70 and Val 73 side chains and the water bridge interactions with the L1 region. The latter is mediated by water molecules between the carboxylate side chain of l-captopril and the backbone amides of Ala 72 and Ala 74. A site-directed mutagenesis study of the F64H variant has also been implicated with increase β-lactam antibiotic resistance (43).

In conclusion, three high-resolution X-ray structures were determined for this investigation and a MD simulation analysis was conducted. Our results demonstrate that Compound **1**, an inhibitor directed against NDM-4, demonstrates a unique binding mode, whereby it forms a bidentate interaction with Zn2 in an octahedral coordination sphere. The data presented herein provides significantly new information for the design of effective inhibitors against the MBLs, which are of particular concern given their increasing dissemination worldwide.

## Experimental procedures

### Protein expression and purification

The starting expression plasmid pET-15B (+)-TEV-NDM-1 (Δ_N1-28_) used in the investigation was a generous gift from the laboratory of Dr Michael W. Crowder, Department of Chemistry and Biochemistry, Miami University. This plasmid was subsequently converted to (Δ_N1-42_) using the Stratagene QuikChange method. Mutation of this construct to generate NDM-4 (M154L variant) was similarly accomplished and utilized to transform Rosetta2(DE3) *E. coli* cells (Novagen) for protein expression. Cultures were grown in lysogeny broth supplemented with 0.1 mM zinc acetate, 100 mg/l ampicillin, and 50 mg/l chloramphenicol.

The cultures were grown at 37 °C with shaking until an absorbance of 0.8 was reached at 600 nm. The flasks were cooled in an ice bath, and the cells were induced with 1 mM isopropyl β-d-1-thiogalactopyranoside and allowed to express protein at 21 °C for 24 h.

The cells were harvested by centrifugation and frozen as pellets in liquid nitrogen. These pellets were subsequently disrupted by sonication on ice in a lysis buffer composed of 50 mM sodium phosphate, 20 mM imidazole, 10% (w/v) glycerol, and 300 mM NaCl (pH 8.0). The lysate was cleared by centrifugation, and the M154L variant was purified at 4 ^°^C utilizing Prometheus nickel-nitrilotriacetic acid agarose (Prometheus Protein Biology Products) according to the manufacturer’s instructions. All buffers were adjusted to pH 8.0 and contained 50 mM sodium phosphate, 300 mM NaCl, and imidazole concentrations of 20 mM for the wash buffer and 300 mM for the elution buffer. The polyhistidine tags were removed by digestion with recombinant tobacco etch virus protease. The recombinant tobacco etch virus protease and remaining tagged proteins were removed by passage over nickel-nitrilotriacetic acid agarose, and the tag-free protein was dialyzed against 10 mM Hepes (pH 7.5) and 100 mM NaCl. The protein was concentrated to 27 mg/ml based on an extinction coefficient of 0.87 (mg/ml)^−1^ cm^−1^.

### Crystallization

Crystals of the apoenzyme were grown *via* the hanging drop method of vapor diffusion at room temperature from 18 to 26% (w/v) poly(ethylene glycol) 3350, 2% (v/v) dimethyl sulfoxide, and 100 mM MES (pH 6.0). They belonged to the monoclinic space group *P2*_1_ with unit cell dimensions of *a* = 39.9 Å, *b* = 59.3 Å, *c* = 41.8 Å, and b = 98.7^°^. The asymmetric unit contained one monomer. For X-ray data collection, the crystals were transferred to a cryo-protectant solution composed of 30% (w/v) poly(ethylene glycol) 3350, 18% (v/v) ethylene glycol, 2% (v/v) dimethyl sulfoxide, and 100 mM MES (pH 6.0).

Crystals of complexes with either l-captopril (Enzo Life Sciences, Inc) or Compound **1** were grown under similar conditions. The protein was first incubated with 10 mM of the respective ligand for ∼30 min before crystallization. Crystals in the presence of l-captopril belonged to the monoclinic space group *P2*_1_ with unit cell dimensions of *a* = 40.1 Å, *b* = 59.3 Å, *c* = 84.2 Å, and β = 98.5^°^. The asymmetric unit contained two monomers. Crystals grown in the presence of Compound **1** belonged to the space group *P2*_1_ with unit cell dimensions of *a* = 39.9 Å, *b* = 59.3 Å, *c* = 41.8 Å, and β = 98.7^°^ and one polypeptide chain per asymmetric unit. Crystals of the enzyme/ligand complexes were prepared for X-ray data collection in the same manner as that for the apoenzyme crystals, except with the addition of 10 mM ligand to the respective cryo-protectant solutions.

### X-ray data collection and processing

All X-ray data were collected at 100 K using a BRUKER D8-VENTURE sealed tube system equipped with HELIOS optics and a PHOTON II detector. The X-ray datasets were processed with SAINT and scaled with SADABS (Bruker AXS). Relevant X-ray data collection statistics are listed in Table 1.

### Structure solution and model refinement

The structure of the apoenzyme was solved *via* molecular replacement with Phaser (49) using PDB entry 5WIG (30) as a search probe. Iterative cycles of model building with COOT (50, 51) and refinement with REFMAC (52) led to a final X-ray model with an overall *R*-factor of 17.9%. Ligand complexes were solved using the apoenzyme structure as a search model. Relevant refinement statistics for all structures are provided in Table 1.

### Kinetic analyses

Nitrocefin (Cayman, CAS 41906-86-9) was used as the chromogenic substrate for all enzyme kinetic assays. The enzymatic activity of purified NDM-1 and NDM-4 was determined spectrophotometrically at room temperature in 1X PBS, pH 7. The rate of product formation was monitored based on the absorbance at 486 nm taken at 10 s intervals for 30 min. The *K*_M_ and *k*_cat_ values were determined from ten different concentrations of nitrocefin ranging from 0.01 μM to 250 μM with at least four independent initial-velocity measurements using 12 nM of each enzyme and fitted by nonlinear regression using Michaelis–Menten enzyme kinetics with GraphPad Prism 9 (https://www.graphpad.com/).

The IC_50_ and *K*_*i*_ values were determined by the direct competition between nitrocefin substrate and inhibitors under appropriately controlled experiments. Different concentrations of inhibitor ranging from 0.001 to 500 μM, a fixed concentration of 12 nM of purified protein, and 20 μM of nitrocefin substrate were used in reaction. The rate of product formation was monitored based on the absorbance at 486 nm taken at 10 s intervals for 30 min. The relative change in absorbance was evaluated as percentage inhibition, and the IC_50_ was determined by fitting the data to a sigmoidal dose-response curve. The enzyme inhibition constant (*K*_*i*_) was derived from initial-velocity measurements by nonlinear regression *via* competitive-inhibition enzyme kinetics using GraphPad Prism 9.

### Computational studies

MD simulations were carried out using Desmond (53). The simulation and analyses details have been previously reported (20, 54). All missing hydrogen atoms were added based on the predicted p*K*_a_ of each ionizable amino acid residues at pH 7 using PropKa (55). Each of structure is solvated inside an orthorhombic box of TIP3P explicit water (56) with a 15 Å buffer region from its outer edge and a default salt concentration of 0.15 M of Na^+^ and Cl^-^ counter ions. Each simulation was initialized with the default minimization, heating, and equilibration protocol, followed by 500 ns production simulation under 310 K and 1 atm constant number of atoms, pressure, and temperature condition using the OPLS3 forcefield (57). The structural stabilities and the fluctuations of the protein backbone were assessed by the C_α__RMSD_ and the C_α__RMSF_. PCA and DCCA based on the C_α_ backbone atom were carried out using bio3D (58) to identify the dominant motion over the course of the simulation and the motion of each region as correlated to one another. Supplemental movies showing each of the PCA movements were generated using visual molecular dynamics movie maker (59).

## Data availability

X-ray coordinates and structure factors have been deposited in the Protein Data Bank under accession codes: 8sk2, 8sko, and 8skp.

## Supporting information

This article contains supporting information.

## Conflict of interest

The authors declare that they have no conflicts of interest with the contents of this article.
