# Supplementary material for: Characterization of a novel inhibitor for the New Delhi metallo-β-lactamase-4: Implications for drug design and combating bacterial drug resistance
Source: J Biol Chem. 2023 Aug 6;299(9):105135. doi: 10.1016/j.jbc.2023.105135 (PMC10514461; doi:10.1016/j.jbc.2023.105135)
Supplement: Supporting Figure S1 [file mmc1.docx]

**Supporting Information**

**Characterization of a novel inhibitor for the New Delhi metallo-β-lactamase-4: implications for drug design and combating bacterial drug resistance**

James B. Thoden^1^, Bogdan M. Benin^2^, Adam Priebe^3^, Woo Shik Shin^2^, Ramaiah Muthyala^4^,

Yuk Yin Sham^4,5,*^, and Hazel M. Holden^1,*^

^1^Department of Biochemistry, University of Wisconsin, Madison, WI 53706

^2^Department of Pharmaceutical Sciences, Northeast Ohio Medical University, Rootstown, OH ^3^Department of Integrative Biology and Physiology, University of Minnesota, Minneapolis, MN

^4^Department of Experimental & Clinical Pharmacology, University of Minnesota, Minneapolis, MN

^5^Bioinformatics and Computational Biology Program, University of Minnesota, Minneapolis, MN

^*^Corresponding authors: Yuk Yin Sham and Hazel M. Holden

Email: shamx002@umn.edu or Hazel_Holden@biochem.wisc.edu

Running title: investigation of the New Delhi metallo-β-lactamase-4

Keywords: antibiotic resistance, antibiotics, computational biology, crystallography, enzyme structure, enzyme kinetics

**­**

**
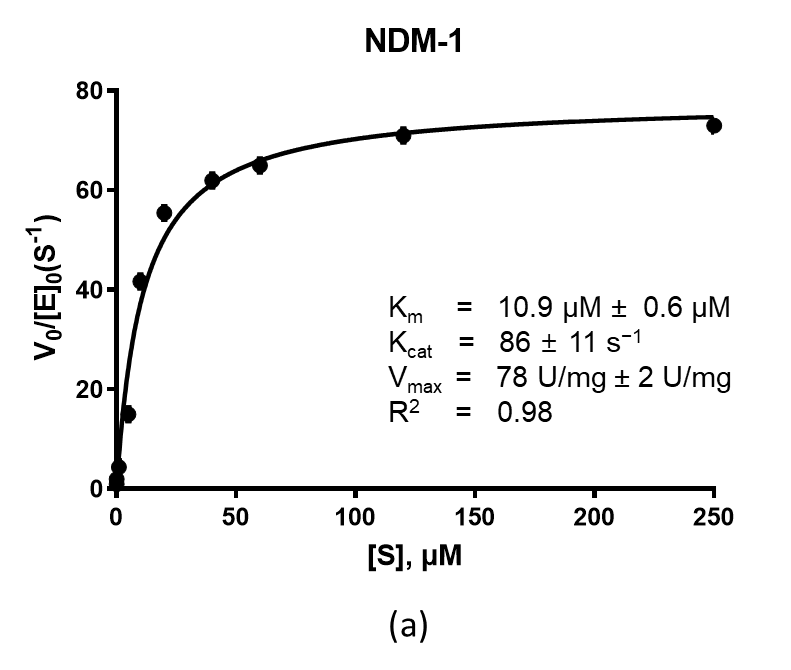
**

**
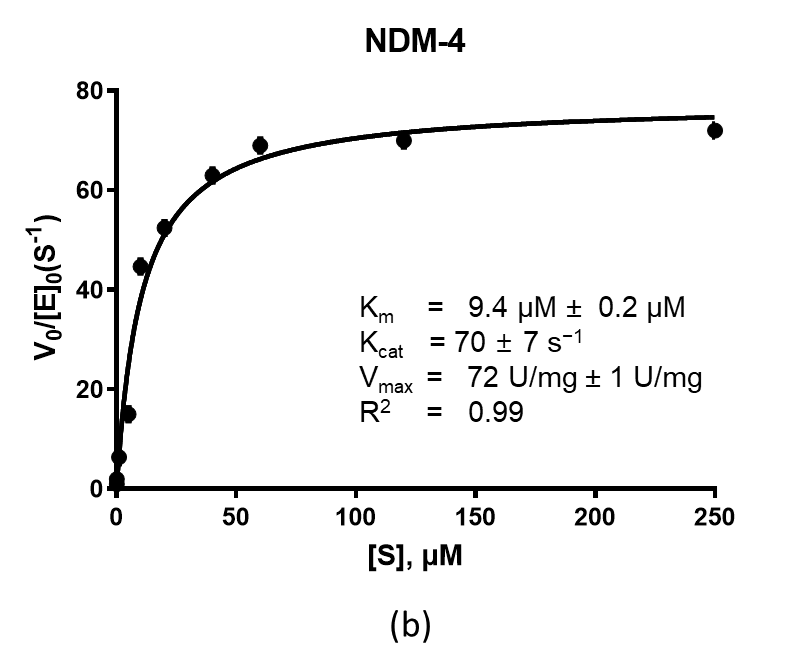
**

**Figure S1.** Steady-state kinetic parameters for NDM1and NDM-4 catalyzed hydrolysis of nitrocefin.
